# Role of Wnt Signaling During In-Vitro Bovine Blastocyst Development and Maturation in Synergism with PPARδ Signaling

**DOI:** 10.3390/cells9040923

**Published:** 2020-04-09

**Authors:** Tabinda Sidrat, Abdul Aziz Khan, Muhammad Idrees, Myeong-Don Joo, Lianguang Xu, Kyeong-Lim Lee, Il-Keun Kong

**Affiliations:** 1 Department of Animal Science, Division of Applied Life Science (BK21 Plus), Gyeongsang National University, Jinju 52828, Korea; tabindasidrat06@gmail.com (T.S.); idrees1600@gmail.com (M.I.); xulianguang428@gmail.com (L.X.);; 2 Institute of Agriculture and Life Science, Gyeongsang National University, Jinju 52828, Korea; 3 Center for Discovery and Innovation, Hackensack University Medical Center, Nutley, NJ 07110, USA; azizkhanuop@gmail.com

**Keywords:** pre-implantation, Wnt/β-catenin, bovine blastocyst, PPARδ, FAO metabolism

## Abstract

Wnt/β-catenin signaling plays vital role in the regulation of cellular proliferation, migration, stem cells cell renewal and genetic stability. This pathway is crucial during the early developmental process; however, the distinct role of Wnt/β-catenin signaling during pre-implantation period of bovine embryonic development is obscure. Here, we evaluated the critical role of Wnt/β-catenin pathway in the regulation of bovine blastocyst (BL) development and hatching. 6 bromoindurbin-3’oxime (6-Bio) was used to stimulate the Wnt signaling. Treatment with 6-Bio induced the expression of peroxisome proliferator-activated receptor-delta (PPARδ). Interestingly, the PPARδ co-localized with β-catenin and form a complex with TCF/LEF transcription factor. This complex potentiated the expression of several Wnt directed genes, which regulate early embryonic development. Inhibition of PPARδ with selective inhibitor 4-chloro-N-(2-{[5-trifluoromethyl]-2-pyridyl]sulfonyl}ethyl)benzamide (Gsk3787) severely perturbed the BL formation and hatching. The addition of Wnt agonist successfully rescued the BL formation and hatching ability. Importantly, the activation of PPARδ expression by Wnt stimulation enhanced cell proliferation and fatty acid oxidation (FAO) metabolism to improve BL development and hatching. In conclusion, our study provides the evidence that Wnt induced PPARδ expression co-localizes with β-catenin and is a likely candidate of canonical Wnt pathway for the regulation of bovine embryonic development.

## 1. Introduction

The development of oocyte into an implantation competent blastocyst (BL) undergoes a series of progressive developmental events. During this process the classical developmental signaling cascades participate to ensure the successful implantation and subsequently improve the pregnancy outcomes [1]. A huge body of evidences has accumulated during the past years that has underlined the importance of Wnt signaling during the pre-implantation period of vertebrate embryonic development [2].

Wnt are a secreted glycoprotein that regulates a plethora of biological functions, including cell proliferation, cell polarity and cell fate determination during embryonic development and tissue homeostasis [3]. The Wnt ligands can activate three distinct intracellular signaling cascades which initiate different biological responses. Canonical Wnt pathway is the classical and well described pathway, which is mediated by transcriptional co-factor β-catenin [3,4]. In canonical Wnt pathway, β-catenin is the key transcriptional co-activator. During the Wnt-off state, the cytoplasmic β-Catenin undergoes a proteasomal degradation by a destruction complex. This degradation leads to the reduced level of cytoplasmic β*-Catenin* [5,6]. The Wnt-on state promotes the disassembly of the destruction complex and prevents the degradation of β-catenin. This leads to the stabilization and translocation of β*-Catenin* into the nucleus, where it binds to TCF/LEF transcription factors and regulates the expression of Wnt target genes [3,7].

Previously, bovine BL microarray analysis has revealed that Wnt signaling components are present during the early developmental stages [8]. A recent study from the bovine embryos also demonstrated the prerequisite of Wnt/β-catenin signaling during the pre-implantation period by the manipulation with Wnt stimulator 2-Amino-4-(3,4-(methylenedioxy)benzylamino)-6-(3-methoxyphenyl) pyrimidine (AMBMP) and Wnt receptor antagonist Dickkopf-related protein 1 (Dkk1) [9]. Another study reported the activation of canonical Wnt signaling as early as the second cell stage of the pre-implantation period, which substantiates the importance of β-catenin dependent Wnt signal transduction [10,11,12,13]. This evidence suggests the role of Wnt signaling during the pre-implantation period. However, how this crucial signaling pathway regulates the development of bovine pre-implantation stages until the BL stage requires further investigation.

Numerous biological responses are triggered either directly or indirectly by Wnt activation [2]. A study by Xie H. et al. demonstrated that during the mouse BL development Wnt couples with peroxisome proliferator-activated receptors (PPARs) signaling and ensure BL competency for implantation. PPAR signaling also regulates the expression of prostaglandin I2 (*PGI2* or prostacyclin) and improves the fatty acid oxidation (FAO) by modulating the genes involved in lipid metabolism [14,15,16]. PPAR signaling is associated with cell proliferation, energy homeostasis, tumorigenesis and metabolic disorders [17,18,19]. These previous studies have all suggested that PPAR is a downstream candidate of Wnt/β-catenin pathway and has a potential role during bovine embryonic development [19,20].

There are several contrasting studies on the role of Wnt signaling during the early period of embryonic development. In one study, it has been shown that the inhibition of Wnt signaling does not compromise the development of embryos to the BL stage. It also claimed that the activation of Wnt signaling by blocking glycogen synthase kinase 3 (Gsk3) activity with LiCl2 or CT99021 had inconsistent effects on development to the BL stage [21]. However, the clear role of Wnt pathway has not been determined to date. Thus, we aimed to investigate the critical involvement of Wnt /β-catenin pathway in synergism with PPAR signaling during the pre-implantation period of bovine embryo. We hypothesized that the complex formed between β-catenin and PPAR with TCF would promote the expression of WNT targeted genes and regulate the BL development. In the present study we stimulated Wnt activity with the small molecule 6 bromoindurbin-3’oxime (6-Bio) a potent agonist of Wnt/β-catenin signaling pathway. The exogenous stimulation of Wnt activity in early bovine embryos up regulated PPARδ expression and promoted PPARδ binding with β-catenin. This binding regulated the expression of several downstream Wnt target genes that, in turn, enhanced the BL development and hatching.

## 2. Materials and Methods

All surgical procedures and experiments were conducted under the rules and legislation approved by the Institute of Animal Care Committee of Gyeongsang National University (Approval ID: GAR-110502-x0017; Date: 02-05-2011).

### 2.1. Drug Treatment for Wnt Stimulation

The 6-Bio (Sigma-Aldrich, Cat # B1686) was used to stimulate in-vitro maturation (IVM) and in-vitro culture (IVC) using Synthetic Oviductal Fluid (SOF) medium. Then, 5 mg powder of 6-Bio was dissolved in dimethylsulfoxide (DMSO) to a concentration of 2.8 mM and stored at −20 °C under light protected condition. Serial dilutions were prepared from stock solution. Cumulus Oocyte Complexes (COCs) were randomly placed in six groups (50-55 COCs per group). 6-Bio was added in each group in 700 µL-volume of culture medium to achieve the final concentration of 0 nM, 200 nM, 300 nM, 400 nM and 500 nM. Based on the bovine BL development and hatching rate, 400 nM dose was selected as the optimal concentration for subsequent experimental analysis. The study was conducted with 8 replicates.

### 2.2. Drug Treatment for PPARδ Inhibition

The 4-Chloro-N-(2-]ethyl)benzamide (Gsk3787) (Sigma-Aldrich, Cat # G7423) was used to specifically inhibit the PPARδ function during the pre-implantation period of bovine embryonic development. A 5 mg powder of Gsk3787 was dissolved in DMSO to make 2.5 mM stock solution and was kept in the dark condition at −20 °C until use. Stock solution was serially diluted in phosphate buffered saline (PBS). COCs were randomly distributed into five groups (n = 50 to 55/group). Different concentrations of Gsk3787 (0 µM, 8 µm, 10 µM, 12 µM and 15 µM) were used in IVM and IVC medium. The study was performed in 10 to 11 reciprocal sets of independent experiments. Based on the blastocyst development and hatching rate, 15 µM dose showed maximum effect on PPARδ activity. For further analysis, 15 µM dose was used.

### 2.3. Aspiration of Oocyte and In-Vitro Maturation

Oocyte aspiration and in-vitro maturation procedure was performed as described previously [22]. Briefly, abattoir collected ovaries were washed with Dulbecco’s phosphate buffered saline (D-PBS). COCs were aspirated in TL-HEPES medium supplemented with 100 IU/mL penicillin, and 0.1 mg/mL streptomycin. Oocytes with compact layer of cumulus cells were collected and washed in TL-HEPES medium. Following washing oocytes were cultured in NUNC 4-well plates containing 700 μL of IVM medium. After 22 h of in-vitro maturation, COCs were in-vitro fertilized with frozen-thawed semen straw.

### 2.4. In-Vitro Fertilization and In-Vitro Culture of Embryos

For in-vitro fertilization, the bovine semen straw was thawed in water bath at 38.0 °C for 1 min and washed in 10 mL DPBS, followed by centrifugation at 750× *g* for 5 min. The pellet formed at the bottom of tube was re-suspended in 10% heparin solution prepared in in-vitro fertilization (IVF) medium (Tyrode lactate solution supplemented with 6 mg/mL BSA, 22 mg/mL sodium pyruvate, 100 IU per mL penicillin and 0.1 mg/mL_1 streptomycin) followed by incubation at 38.0 °C in a humidified atmosphere containing 5% CO_2_ for 15 min. Spermatozoa was diluted with IVF medium with a final concentration of 1 × 10^6^ per mL. For each group 50 to 55 matured COCs were transferred into a 4-well dish and fertilized with 700 μL of IVF medium. These dishes were incubated in a humidified condition with 5% CO_2_ at 38.0 °C for 18–20 h. After fertilization, oocytes were cleared from the cumulus cells by repeated pipetting and denuded presumptive zygotes were cultured for 8 days. BLs were cultured in a SOF media supplemented with 44 μg/mL sodium pyruvate (C3H3NaO3), 14.6 μg/mL glutamine, 10 IU/mL penicillin, 0.1 mg/mL streptomycin, 3 mg/mL FBS and 310 μg/mL glutathione. At day-8, BLs were washed three times in 1 × PBS and stored at 4 °C after fixation in 4% paraformaldehyde until further analysis. For gene expression analysis, BLs were immediately snap-frozen in liquid nitrogen after washing in nuclease free water and stored at −80 °C in 1.5-mL Eppendorf tubes.

### 2.5. RNA Extraction and Quantitative Real Time PCR (q-RT-PCR) Analysis

Total RNA from BLs (n = 5 per group) was isolated using RNA isolation kit (PicoPure, ThermoFisher). Subsequently, cDNA synthesis and quantitative real time polymerase chain reaction (q-RT-PCR) was performed by using iScript Reverse transcriptase (BioRad, Cat # 1708891) and SYBR Green master mix (BioRad, Cat # 170-8882AP), respectively. PCR amplification was performed with the following conditions: initial denaturation at 94 °C for 5 min followed by 40 cycles of 94 °C for 30 s, 58 °C for 30 s and 72 for 30 s. Relative mRNA expression of all genes was normalized with *GAPDH*. For the mRNA expression analysis, experiment was performed in triplicate sets. Primers used for qRT-PCR are listed in Table S1.

### 2.6. Immunofluorescence Analysis

Immunofluorescence staining was performed as described previously [22]. Briefly, different developmental stage embryos and BLs were fixed with 4% (*w/v*) PFA (paraformaldehyde) solution, and permeabilized with 0.5% Triton X-100. After blocking, sequentially incubated with primary antibody overnight and secondary antibody either conjugated with FITC or TRITC for 1 h at RT. Samples were washed extensively with PBS/polyvinylpyrrolidone (PVP) and the nuclei were counterstained with DAPI for 5 min at RT. Images were viewed under a confocal laser microscope (Olympus, FV1000). The antibodies used for immunofluorescence are listed in Table S2.

### 2.7. Quantification of Lipid Content by Fluorescent Probe Nile Red 

To analyze the accumulation of lipid droplets, a fluorescent probe (Nile red, NR) specific for intracellular lipids was used to evaluate the lipid content in the day-8 fixed BLs. NR stock solution was prepared by dissolving 1 mg/mL in DMSO, stored at room temperature under dark condition. Day-8 BLs were fixed in 4% (*w/v*) PFA solution. BLs were washed three times with PBS and incubated with PBS/PVP containing 10 mg/mL NR solution for 3 h at room temperature in protected light condition. Stained BLs were washed 3 times with PBS/PVP solution and incubated with DAPI (1:100 (*v*/*v*) in PBS/PVP for 5min. BLs were mounted on a glass slide with an overlaid cover slip after washing twice with PBS. Thereafter, the NR lipophilic fluorescent dye (485 nm) was excited under confocal laser-scanning Olympus FluoviewFV1000 microscope. Red fluorescence (lipids) intensities were measured by using Image J (National Institute of Health, Bethesda, MD, USA) software. Normalization was performed by subtracting the background intensity from each image of the experimental groups. The lipid contents from each group is presented as mean fluorescence intensity.

### 2.8. Invasion Assay

To quantify the effect of Wnt pathway stimulation on BL implantation potential, the spreading and invasion areas of day-8 BLs were quantified. Blastocysts were placed on an invasion chamber insert (6.4 mm; Corning Inc. Life Sciences) containing polyethylene terephthalate membranes (8 µm-diameter pores) in 24-well tissue culture plates (Corning Inc. Life Sciences). The upper surface of the chamber was coated with Matrigel (20 mg per filter; Discovery Labware Inc.) and incubated for 2 h at 37 °C for drying. Three BLs per culture insert were placed in the Matrigel coated chamber containing the same medium used for embryo culture. Incubated at 37 °C for 72 h, replaced with fresh medium every 24 h at the bottom of the culture chamber. The invasion and spreading area of trophoblasts after 10 days of culture was examined under a phase contrast Olympus IX71microscope and analyzed using Image J software. Thereafter, a cotton swab was used to scrub the upper surface of the chamber insert membrane. Cells were fixed with 4% (*w/v*) paraformaldehyde on the lower surface of the scrubbed membrane for 15 min at room temperature. Then stained with DAPI for 5 min. Cells that passed across the membrane were counted under a phase-contrast Olympus IX71 microscope.

### 2.9. Statistical Analysis

Statistical analysis was performed using SPSS software version 18.0 (IBM Corp., Armonk, NY, USA). All percentage data are presented as mean ± standard error of mean (SEM). Triplicate sets of experiment were used to obtain all the image data, and single BL image was shown as representative image from the individual group, whereas all graphical data obtained from triplicate sets of experiments are presented as the mean standard error of mean (SEM). All mean fluorescence intensities presented in the present study were quantified per BL from each group, *n* = 20 BLs per group from individual experiment. Histogram values of fluorescence intensities were measured by Image J software (USA). All results for the expression level of various genes or comparison of fluorescence intensities were analyzed by using one-way analysis of variance followed by Sidak’s Multiple Comparison Test. GraphPad Prism 6.0 software package (USA) was used. Significant differences were considered at * p < 0.05; ** p < 0.01; *** p < 0.001.

## 3. Results

### 3.1. Augmentation of Canonical Wnt Activity via 6-Bio Treatment Enhances Bovine BL Development and Hatching

**Figure 1:**
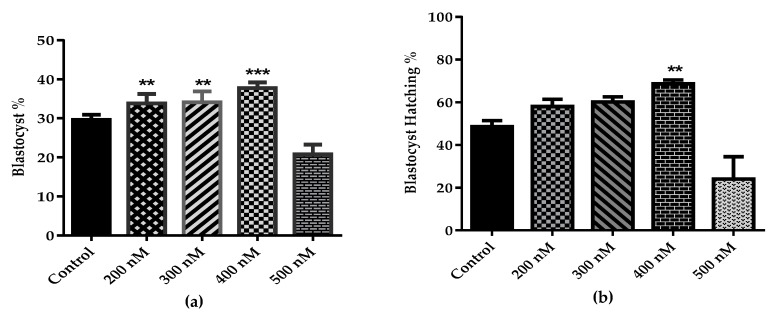

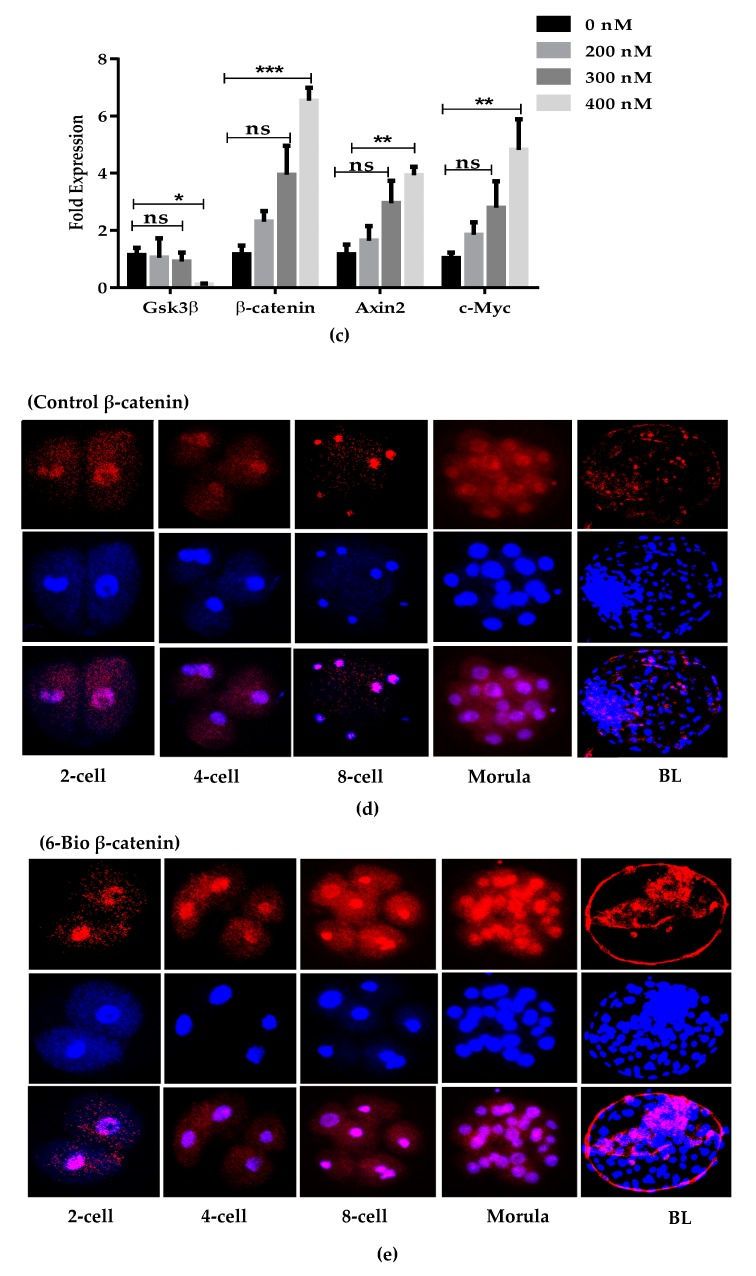
Effect of 400 nM dose of 6-Bio treatment on Wnt-directed genes expression and bovine embryonic development. (**a**,**b**) Representative bar graph showing the % of BLs development and hatching rate after addition of different concentrations of Wnt agonist 6-Bio. Data are presented as a result of ± standard error of mean (SEM) of 8 replicates. (**c**) qRT-PCR analysis of the expression profile of several Wnt components in response to the indicated concentrations of 6-Bio treatments. (**d**–**e**) Immunofluorescence analysis showing the expression of activated β-catenin in control versus 6-Bio treated group during pre-implantation stages. DAPI staining (blue; all nuclei). * *p* < 0.05; ** *p* < 0.01; *** *p* < 0.001 indicates significant difference. Original magnification 100×.

To evaluate the stimulatory effect of specific agonist of Wnt/β-catenin pathway, we used 6-Bio during in-vitro maturation and culturing of bovine embryonic development. First, we determined the optimal dose of 6-Bio for maximal activation of Wnt signaling pathway. Exogenous stimulation of Wnt activity with 6-Bio dose improved the bovine BL formation and hatching rate (Table 1). The 400 nM concentration of 6-BIO showed the highest BL development and hatching rate (Figure 1A,B). qRT-PCR confirmed the expression of several well-known canonical Wnt target genes in the bovine BL. The expression of target genes was increased by the addition of 6-Bio in a dose dependent manner (Figure 1C). Treatment with 6-Bio significantly enhanced the β-catenin expression during the pre-implantation stages in comparison to the embryos grown in the un-stimulated Wnt condition. There was a significant difference among all stages (Figure 1D,E, Figure S1). These results corroborated that exogenous activation of the canonical Wnt pathway by 6-Bio significantly improves the bovine embryonic development.

### 3.2. Effect of 6-Bio Treatment on Proliferation and Lineage Specification during BL Development

**Figure 2:**
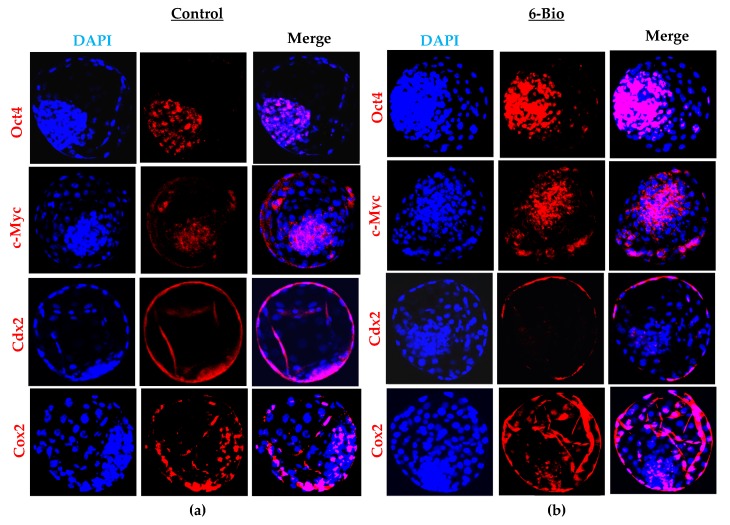

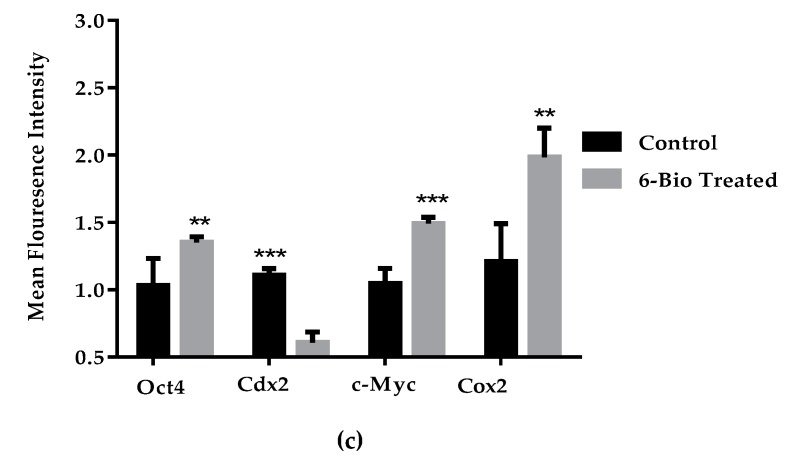
Wnt role in pluripotency and lineage specification during bovine BL development. (**a**,**b**) Representative images of pluripotency and lineage specific markers in control and 6-Bio (400 nM) treated group. DAPI labeled nuclei in blue. (**c**) Bar graph data of mean fluorescence intensities presented as a means ± SEM from three independent sets of experiment, including n = 20 BLs per group in each replicate. ** *p* < 0.01; *** *p* < 0.001 indicates significant difference. Original magnification 100×.

The functional importance of the Wnt signaling pathway during bovine embryonic development was characterized by analyzing several pluripotency and lineage specification markers. The expression of pluripotency maker Oct4 displayed a significant up regulation in the inner cell mass in the 6-Bio treated group (Figure 2A,B). Similarly, c-Myc, a target of the Wnt/β-catenin pathway was up regulated upon Wnt activation in the inner cell mass and trophectoderm of the bovine blastocyst (Figure 2A,B). The expression of trophoblast transcription factor, Cdx2 was downregulated by Wnt activation in the 6-Bio treated BL within the inner cell mass and trophectoderm layer. Cyclooxygenase-2 (Cox2) which promote the growth and hatching of the BL showed pronounced increase with the 6-Bio treatment (Figure 2A,B). qRT-PCR further confirmed the immunostaining analysis (Figure 2C). All these observations show the importance of Wnt signaling during the development of bovine embryos. It also suggests that the activation of Wnt signaling may exert a positive effect on the maintenance of pluripotency marker genes and may regulate the early stages of development.

### 3.3. Exogenous Stimulation of Canonical Wnt Activity Induces PPARδ Signaling during Bovine Embryonic Development

**Figure 3:**
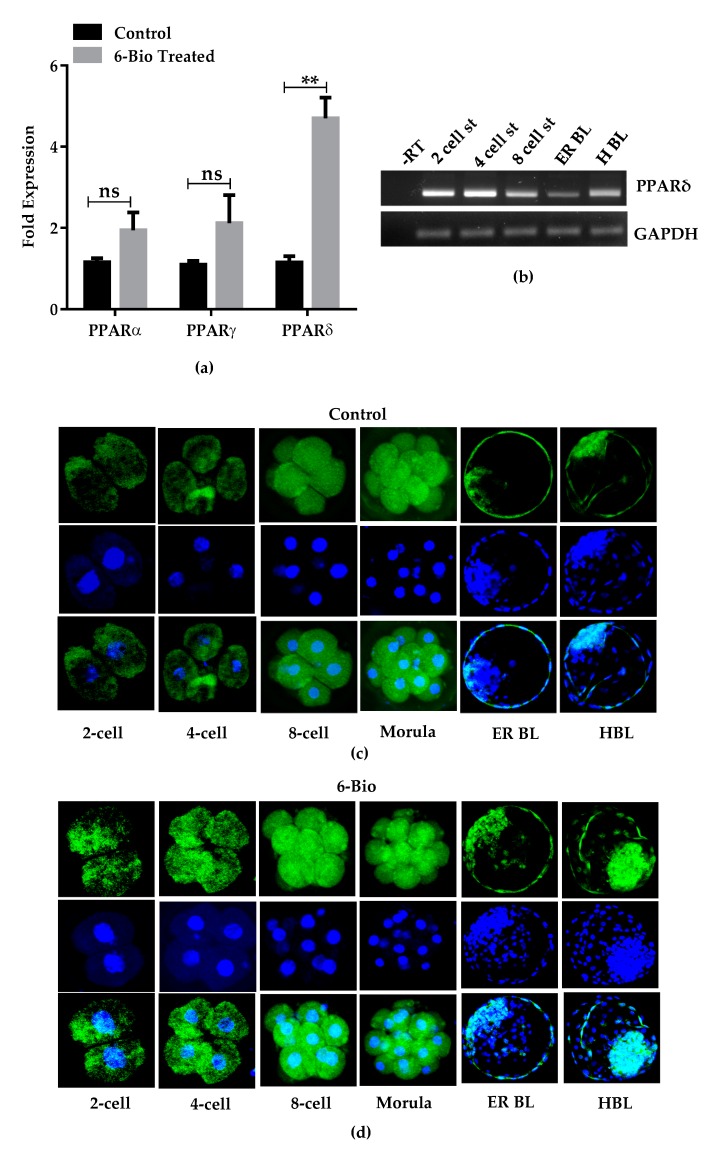
PPARδ expression in response to Wnt activation during bovine embryonic development. (**a**) Relative mRNA expression analysis of *PPAR* family in response to Wnt stimulation with its agonist 6-Bio (400 nM). n = 5 BLs, experiment performed in triplicate sets. ns; non-significant, ** *p* < 0.01; indicates significant difference. (**b**) RT-PCR displaying the PPARδ expression during the time course of bovine embryonic development. (**c**,**d**) Immunofluorescence analysis showing the concomitant increase in the PPARδ expression during different stages of bovine pre-implantation development in 6-Bio (400 nM) treated group and control group. Original magnification 100×.

To further explore the role of Wnt/β-catenin pathway in the bovine embryonic development, we probed the expression of genes that are influenced by the Wnt/β-catenin pathway. The exogenous stimulation of Wnt activity strongly enhanced the expression of Cox2. Cox2 is a key enzyme in the production of prostaglandins and plays an important role in number of reproductive processes [23,24]. Several cancer studies reported that the elevated expression of Cox2 is associated with increased PPAR expression [17,25]. The increase in the expression of Cox2 in bovine BL stage led us to hypothesize that PPAR genes can be strongly influenced by Wnt/β-catenin pathway during the pre-implantation period of bovine embryos. There are no pronounced data available that show the expression and functional analysis of three isoforms of PPARs family. This led us to probe the expression of PPAR genes in response to Wnt stimulation. We performed qRT-PCR analysis with BL cultured in the Wnt activated condition. Among the three members of PPAR family, the expression of PPARδ was predominantly enhanced upon Wnt stimulation (Figure 3A). By RT-PCR analysis we detected the PPARδ expression in different stages of bovine embryonic development (Figure 3B). Furthermore, immunofluorescence analysis determined that bovine embryos cultured in media supplemented with Wnt agonist had increased PPARδ expression during all developmental stages of pre-implantation period (Figure 3C,D). All these results suggested that PPARδ is positively regulated by Wnt signaling pathway and these two synergistically regulate the bovine embryogenesis.

### 3.4. Wnt Regulate BL Development by Sustaining Proliferative Signaling via Modulating PPARδ Expression

**Figure 4:**
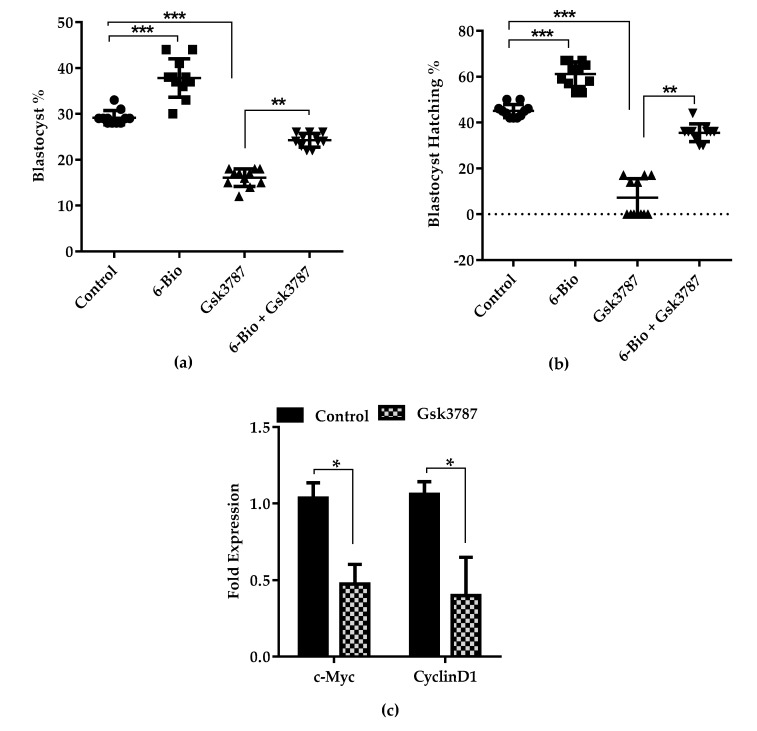

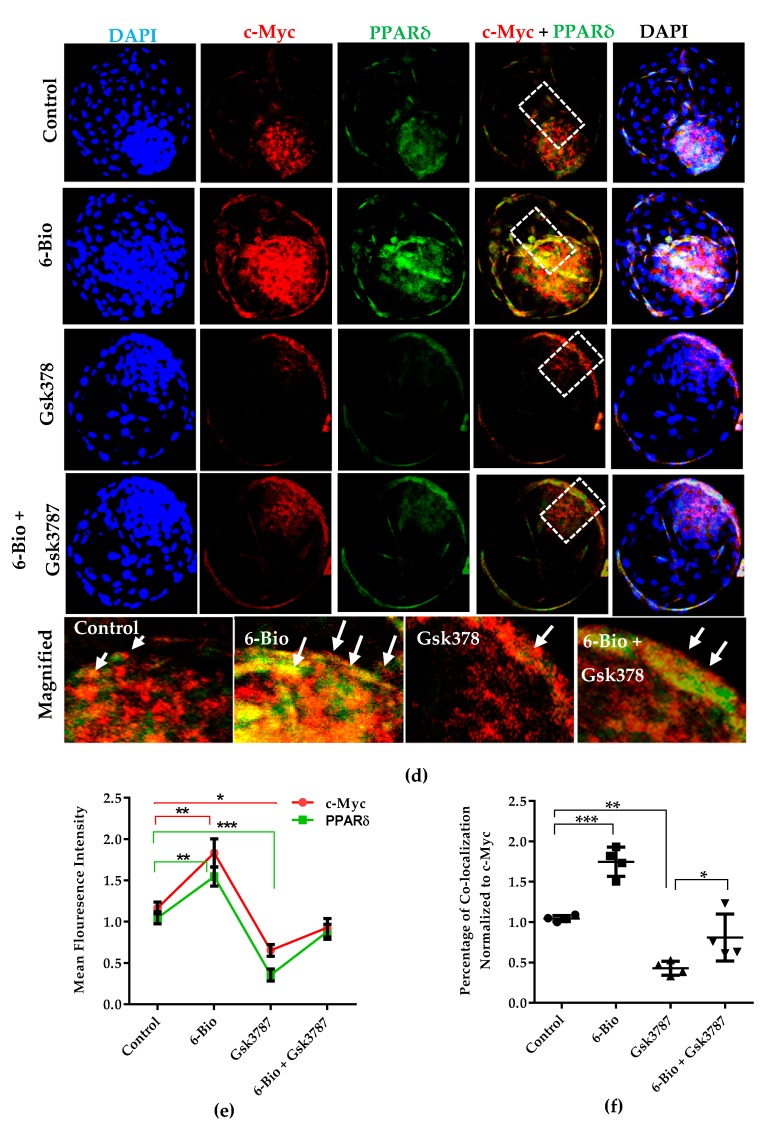
Wnt induced PPARδ signaling upregulates c-Myc expression during bovine BL development. (**a**) Bar graphs represents the treatment of Wnt agonist (6-Bio; 400 nM), PPARδ antagonist (Gsk3787; 15 µM) and their co-treatment effect on bovine embryonic development to the BL stage. Data presented as a result of ± standard error of mean(SEM) from 11 replicates. (**b**) qRT-PCR analysis of c-Myc and CyclinD1 expression by Gsk3787 treated group. n = 5. (**c**) Immunofluorescence imaging showing co-localization of PPARδ (green) with c-Myc (red) in response to 6-Bio stimulation. Arrows indicate overlapping of PPARδ and c-Myc expression merge in Yellow. Nuclei counterstained with DAPI (blue). Higher magnification of the box regions shown at the bottom (**d**,**e**) Bar graph represent the quantification of mean fluorescence intensities and percentage of co-localization of PPARδ with c-Myc. All data are mean ± SEM from three independent sets of experiments (n = 20 BLs). * *p* < 0.05; ** *p* < 0.01; *** *p* < 0.001 indicates significant difference. Original magnification 100×.

PPARδ expression is documented to be highly associated with Wnt signaling [26]. Carcinogenesis status can be altered by the modulation of PPARδ expression with the use of specific agonist and antagonist [27,28]. Based on early findings, we hypothesized that cell proliferation in the embryonic preimplantation development is regulated by the Wnt signaling pathway through the mediation of PPARδ expression. To substantiate this hypothesis, we blocked the function of PPARδ with specific inhibitor Gsk3787. The treatment of Gsk3787 attenuated the PPARδ function and alter the blastocyst formation rate, predominantly the quality and hatching of BL in a dose dependent manner (Table S3, Figure S2A,B). This antagonistic effect of Gsk3787 was well rescued by 6-Bio treatment (Table 2, Figure 4A,B). These results suggest that elevated level of PPARδ expression in response to Wnt agonist may synergizes with Wnt/β-catenin signaling and improve the bovine BL development and hatching. Furthermore, we analyzed the effect of PPARδ antagonist Gsk3787 on cell proliferation index. Blocking of PPARδ significantly reduced the cell proliferation ratio and impaired the quality of BL whereas the inhibitory effect of PPARδ antagonist was rescued upon the addition of Wnt agonist (Figure S3). Next we probed the effect of PPARδ inhibition on Wnt targeted cell proliferation markers i-e c-Myc and CyclinD1. The expression of these genes was significantly reduced in Gsk3787 treated group relative to the untreated group (Figure 4C). Based on the above observations we hypothesized that PPARδ expression may co-localizes with Wnt directed genes expression and promote the cell proliferation event during BL development. BLs cultured in Wnt agonist media showed the co-localized expression of c-Myc with PPARδ as shown by immunofluorescence analysis. Whereas the expression of both c-Myc and PPARδ was significantly reduced in the group cultured in the presence of Gsk3787 inhibitor. The reduced expression was successfully rescued in the group supplemented with Gsk3787 in combination with 6-Bio (Figure 4D–F). These results suggested that the Wnt/β-catenin signaling up-regulate the PPARδ signaling which synergistically promote the cellular proliferation and enhances the developmental potential of the bovine BL.

### 3.5. Wnt Promote BL Development and Hatching via Regulating FAO Metabolism through PPARδ Signaling 

**Figure 5:**
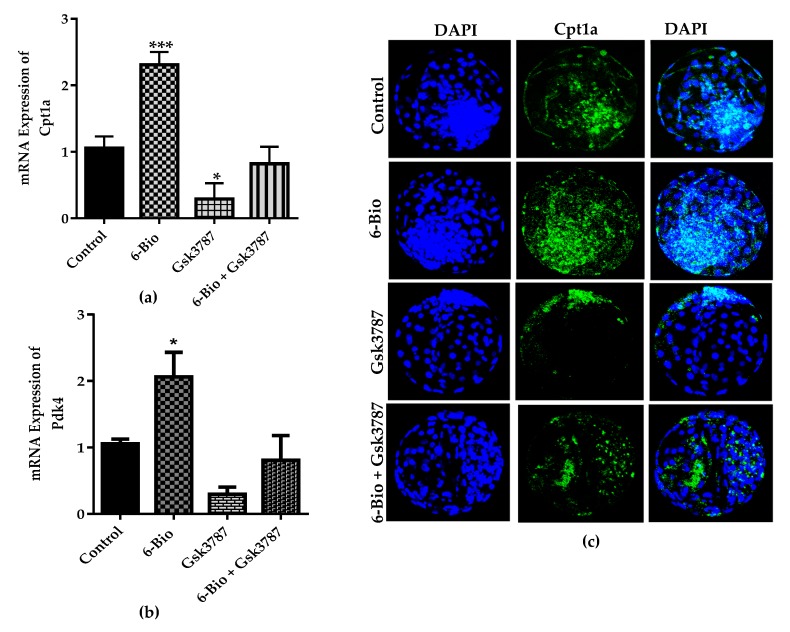

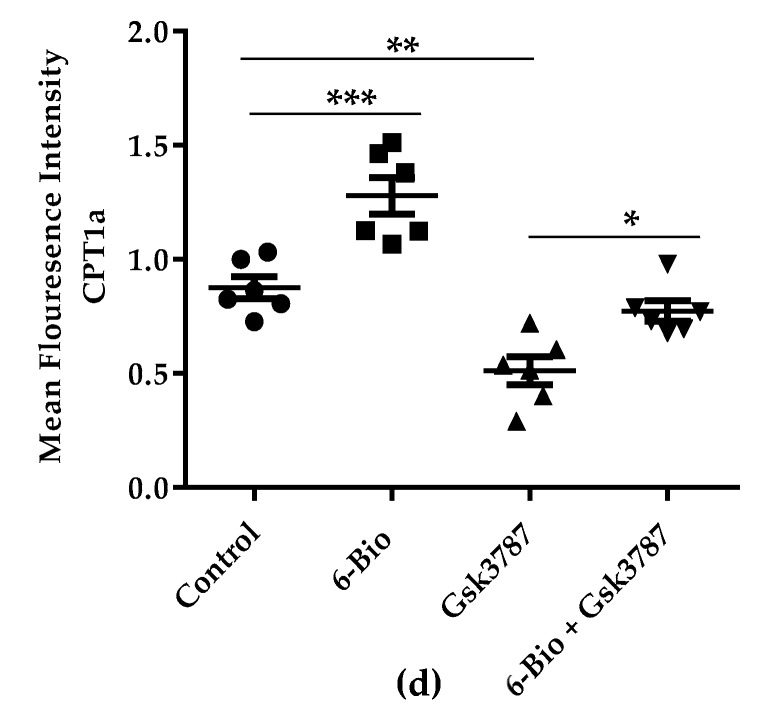
Wnt stimulate fatty acid oxidation (FAO) metabolism through PPARδ-Cpt1-axis to regulate BL development and Hatching. (**a**,**b**) qRT-PCR analysis for FAO regulating genes Cpt1 and Pdk4 expression by 6-Bio (400 nM), Gsk3787 (15 µM) and 6-Bio + Gsk3787 treated groups. n = 5 BLs. (**c**) Immunofluorescence imaging showing Cpt1 expression. DAPI labeled nuclei in blue. (**d**) Bar graph data of mean fluorescence intensities presented as a means ± SEM from three individual sets of experiment, including n = 20 BLs per group in each replicate. * *p* < 0.05; ** *p* < 0.01; *** *p* < 0.001 indicates significant difference. Original magnification 100×.

In the early embryonic development, the cell proliferation event is accompanied by several metabolic process. The most important of these metabolic processes is the lipid metabolism [29]. The ATP is generated by the fatty acid oxidation (FAO) during the early embryonic development and BL hatching [30]. Moreover, extensive reports show that PPAR family of transcription factors are known to regulate the expression of several key factors involved in FAO [15,30,31]. Based on these observations, we hypothesized that Wnt pathway may accelerate the BL hatching by the induction of PPARδ expression and FAO metabolism. We subsequently examined the expression of Cpt1 and Pdk4. These are the key regulatory genes involved in FAO and are the downstream targets of PPAR family of transcription factors. qRT-PCR analysis revealed that stimulation of Wnt significantly induced the expression of Cpt1 and Pdk4. Blocking of PPARδ function with Gsk3787 strongly reduced the expression of FAO related genes. The expression of these genes was restored by combination of Gsk3787 and 6-Bio (Figure 5A,B). Furthermore, the antagonistic effect of PPARδ inhibitor on Cpt1 expression was also confirmed by immunofluorescence analysis. These observations were consistent with qRT-PCR analysis (Figure 5C,D). These results suggested that Wnt signaling promote FAO metabolism by the upregulation of PPARδ signaling and accelerate BL hatching rate.

### 3.6. Canonical Wnt Activation in Co-ordination with PPARδ Reduces Lipid Content and Enhances BL Implantation Potential

**Figure 6:**
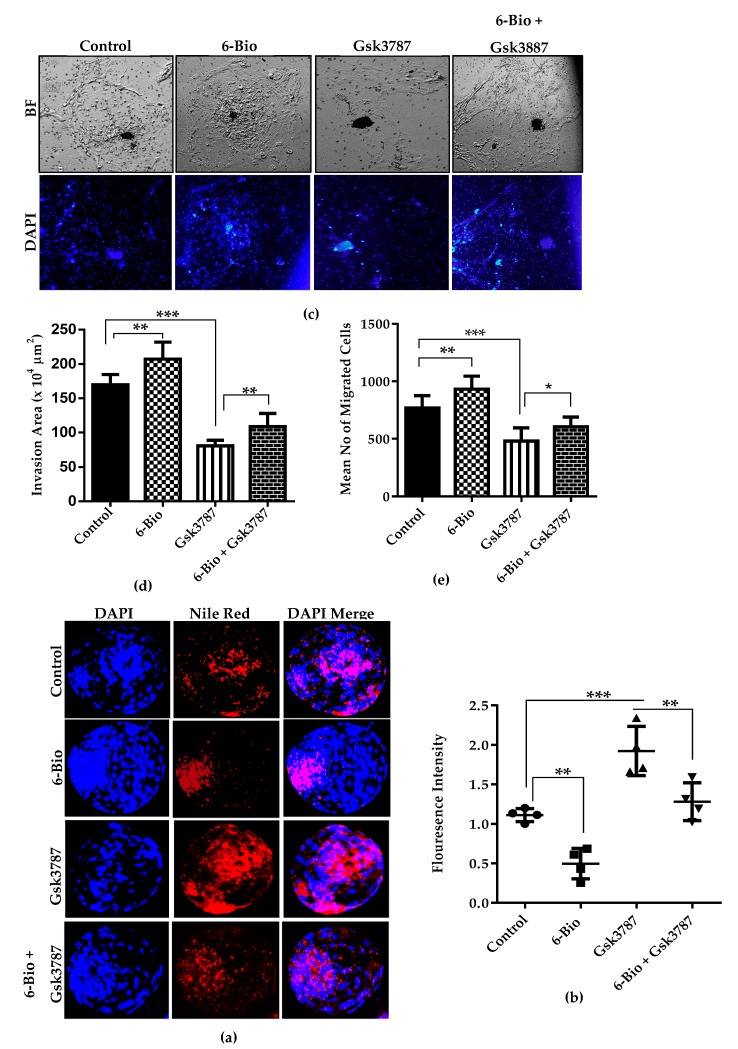
The effect of Wnt activation on lipid accumulation and invading ability of BL trophectoderm cells. (**a**) Fluorescent Nile probe (red) staining in control, Wnt agonist (400 nM), PPARδ antagonist (15 µM) and combination of agonist + antagonist treated groups. (**b**) Quantification of mean fluorescence intensities shown in the graph. (**c**) Bright field images showing the invasion area of BLs cultured in 6-Bio, Gsk3787 and 6-Bio + Gsk3787 supplemented medium. (**d**) Representative bar graph shows the mean invasion area analyzed by image J software. (**e**) Bar graph present the mean number of migrated cells. Nuclei are stained in blue in all images. Data presented as means ± SEM from triplicate sets of experiment, including n = 20 BLs per group in each replicate. * *p* < 0.05; ** *p* < 0.01; *** *p* < 0.001 indicates significant difference. Original magnification 100×.

Our investigations showed that Wnt stimulated condition enhanced the lipid metabolism by up regulating PPARδ expression. To better understand the categorical role of Wnt/β-catenin signaling on lipid metabolism, we assessed the lipid droplets accumulation by Nile red staining during BL development. The immunostaining analysis displayed that the accumulated lipid content is profoundly low in 6-Bio treated group as compared to control group. PPARδ antagonist (Gsk3787) treated group exhibited a significant increase in the lipid droplets accumulation. However, treatment with Wnt agonist 6-Bio in combination with Gsk3787 reduced the lipid droplet content in the developing BL (Figure 6A,B). These results suggest that the dramatic reduction in lipid droplet content during bovine embryonic development might be due to high FAO metabolism in response to Wnt induced PPARδ activity. The high FAO metabolism may improve the BL development and hatching rate to efficiently meet the high energy demand during the pre-implantation period.

We also analyzed the effect of activated Wnt condition on BL competency for implantation. During the pre-implantation development, the BL achieves the competency to attach to the uterus. This attachment is essential for a normal and successful implantation process [2]. To gain further insight into canonical Wnt signaling induced PPARδ expression we exploited the activator and the inhibitor approach. The effect was observed by the invasion assay. BL cultured in Wnt activated condition was able to spread on a significantly enlarged area with respect to control BL. The inhibition of PPARδ led to weak BLs attachment and reduced cell invading ability. This effect was reversed upon the combined treatment of 6-Bio with Gsk3787 (Figure 6C). Furthermore, quantification of mean invasion area and the number of migrated cells showed a significant difference between control and drug treated groups (Figure 6D,E). These observations demonstrate that Wnt/β-catenin activity requires coordination with PPARδ signaling to regulate bovine BL implantation process.

### 3.7. Exogenous Stimulation of Canonical Wnt Activity Promote Co-localization of PPARδ with β-Catenin during Bovine Embryonic Development

The stimulation of Wnt/β-catenin activity with its agonist directly induces the expression of PPARδ in bovine embryos. This stimulation in turn influences the pre-implantation period of development and BL hatching rate by mediating the expression of direct Wnt/β-catenin targeted genes. There are many conflicting studies regarding the functional interaction of PPARδ with Wnt/β-catenin pathway. β-catenin is a transcriptional co-activator of TCF/LEF transcription factor, which triggers the expression of several genes regulated by canonical Wnt signaling pathway [4,32]. We were interested to investigate the interaction between PPARδ and β-catenin during bovine BL development. 

**Figure 7:**
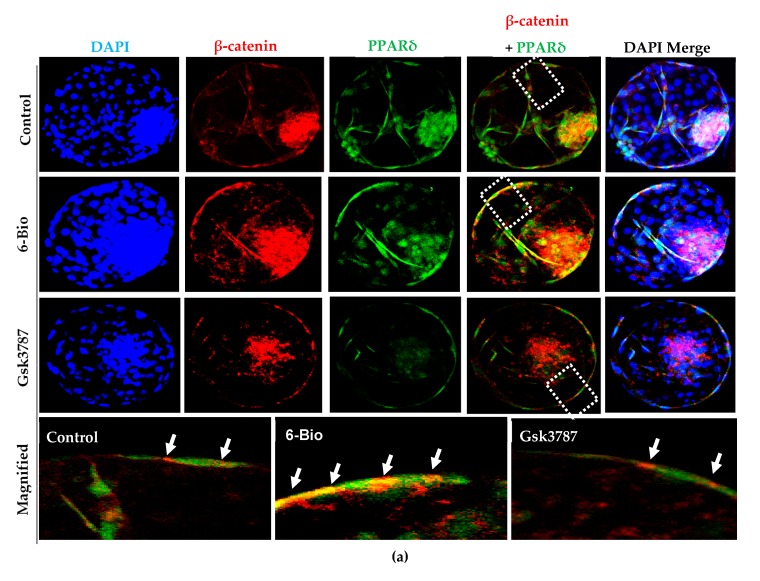

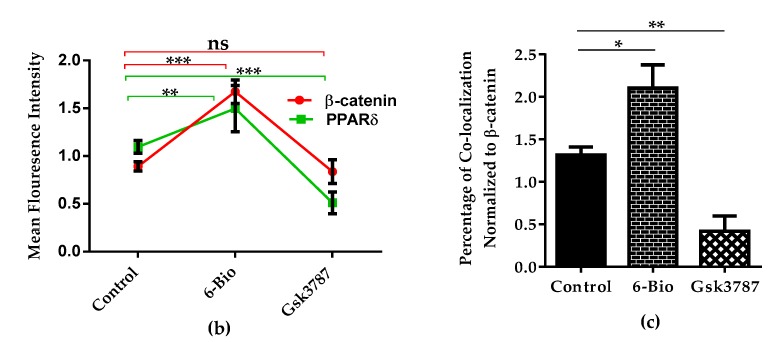
PPARδ co-localizes with β-catenin upon Wnt stimulation to regulate bovine embryonic development. (**a**) Immunofluorescence imaging showing co-localization between PPARδ (green) and β-catenin (red) is strengthened upon Wnt stimulation. Arrows indicate overlapping of PPARδ with β-catenin expression merge in (yellow). Nuclei counterstained with DAPI (blue). Higher magnification of the box regions shown at the bottom (**b**,**c**). Bar graph represents the quantification of mean fluorescence intensities and percentage of co-localization of PPARδ with β-catenin. All data are mean ± SEM from three individual sets of experiments (n = 20 BLs). * *p* < 0.05; ** *p* < 0.01; *** *p* < 0.001 indicates significant difference. Original magnification 100×.

The stimulation of Wnt activity strongly enhanced the spatial overlapping of PPARδ with β-catenin while PPARδ was randomly overlapped with β-catenin in control group as shown by immune fluorescence analysis (Figure 7A). This result is consistent with previous study which showed that PPARδ can interact with β-catenin and promote Wnt/β-catenin mediated human colorectal cancer progression [19]. Reversibly, inhibition of PPARδ with Gsk3787 treatment significantly reduced its co-localization with β-catenin. The expression was restored eventually by the addition of 6-Bio (Figure 7A–C). The quantification analysis for the β-catenin and PPARδ expression positive cells has been shown in (Figure S4A,B). These results illustrated that Wnt upregulated the expression of PPARδ and enhanced its co-localization with β-catenin. This co-localization may make a transcriptional co-activator complex to promote the Wnt targeted genes expression and regulate bovine BL development.

## 4. Discussion

During the pre-implantation development, the subsequent formation of distinctive embryonic lineages is governed by the activation of the specific signaling pathways [3]. Wnt is one of the pioneers in the developmental signaling cascade study. It is known to play an important role in the regulation of several developmental processes, such as pluripotency maintenance, embryonic axis formation, cell proliferation and migration during the pre-implantation period of vertebrate embryos [4]. However, the exact role of Wnt signaling pathway for regulation of pre-implantation development of bovine embryo is not well known. Some studies reported that Wnt pathway is active in bovine preimplantation embryos [8,9], but the biological function of Wnt activation during bovine BL development has not been well investigated. 

Our present investigation using small-molecule agonist (6-Bio) of canonical Wnt pathway provides an evidence that activation of Wnt/β-catenin signaling is important during the pre-implantation period of in-vitro produced bovine embryos. The reciprocal stimulation with 6-Bio treatment significantly improved BL development and hatching rate (Figure 1A,B). 6-Bio treatment inhibited the Gsk3β expression in the bovine BL which consequently stabilizes the expression of β-catenin (Figure 1C–E). This result is consistent with the early findings which showed that inhibition of Gsk3β activity led to the accumulation of active β-catenin with improved embryo quality [21], and provided the evidence of positive co-relation between Wnt signaling and normal bovine embryo development. On the other hand, the hyper activation of Wnt signaling pathway with 500 nM concentration of 6-Bio hampered the embryonic development and reduced the BL formation rate (Figure 1A,B). In parallel to the main effector of Wnt pathway, β-catenin is also a main component of the adherens junction assembly [8,13]. It has also been postulated that nuclear β-catenin is required at certain threshold level, reaching that level above might unbalance the amount of nuclear versus adherens junction complex β-catenin and leads to the loss of cell-cell adhesion [33], perhaps which prevent the proper differentiation of the cell. The underlying mechanism of this phenomenon needs further investigation. 

During early embryo development, the maintenance of cell proliferation event is regulated by the activation of several pluripotency factors and their interaction with the Wnt signaling cascade [34,35,36,37]. In this study exogenous stimulation of 6-Bio significantly induced the expression of core pluripotency markers such as Oct4 and c-Myc (Figure 2). The up regulation of these genes might help in the maintenance of pluripotency of ICM cells during BL formation. In parallel with the activation of Oct4 and c-Myc, the elevated β-catenin signaling suppressed the Cdx2 expression domain in the trophectoderm cells of developing BL (Figure 2). It has been reported previously that Oct4 and Nanog are required to suppress the expression domain of Cdx2 in the ICM cells [38,39]. Thus, in our results the significant up regulated expression of Oct4 protein in the ICM cells gives the possible explanation for the detectable loss in Cdx2 expression within the ICM. Although the Cdx2 expression was lost in 6-Bio treated embryos but it did not impair the BL formation rate. This might be due to the expression of upstream factors or of parallel to Cdx2 genes such as Tead4, Yap1, Gata3 and Elf5. These factors are adequately expressed in bovine trophoblast cells and initiates the trophectoderm differentiation [40,41,42]. 

Different cancerous and developmental studies reported the cross-talk between Wnt/β-catenin pathway and the nuclear hormone receptor PPARs family [19,43]. PPARδ has been observed to regulate the BL development and implantation process in the mice uterus in response to Cox2 activation [44]. Here, we also observed that 6-Bio treatment remarkably increased the Cox2 expression (Figure 2). This result provided a link between PPARδ expression and Wnt signaling during bovine BL development. We demonstrated that activation of Wnt/β-catenin pathway by 6-Bio results in significant induction of PPARδ expression among the other PPAR family members (PPARα, PPARγ) (Figure 3A–D). These results affirmed the importance of Wnt induced PPARδ expression for the regulation of bovine BL development. Furthermore, we showed that PPARδ works in conjunction with Wnt activation to enhance the cell proliferation activity via stabilizing the strong co-localization with direct Wnt target gene c-Myc (Figure 4D), whereas its inhibition with GSK3787 decreased cell proliferation ratio. These results highlighted the necessity of PPARδ expression during Wnt activation and suggests that decreased cell proliferation activity interfere with the development of embryos to reach to the BL stage. 

PPARδ is critically involved in regulation of lipid metabolism and positively influence the BL hatching [15]. Interestingly, PPARδ inhibition during pre-implantation development of bovine embryos caused the down regulation of two major FAO regulating genes Cpt1 and Pdk4. The expression of these genes was restored in the presence of Gsk3787 and 6-Bio containing media (Figure 5). Consistent with this observation, more accumulated lipid droplets were found in the bovine BL group treated with selective PPARδ antagonist. This effect was reversed upon the addition of Wnt agonist (Figure 6A). Lipid droplets are linked to energy balance and facilitates ATP production via Cpt1, a key enzyme involved in FAO during early embryonic development [45]. Based on our results and previous reports, we predicted that the upregulation of Cpt1 and Pdk4 preferentially utilizing the fatty acids during in-vitro development and growth. This observation is further strength by the less accumulation of lipid content in the presence of 6-Bio. Moreover, during the BL implantation process, the implication and necessity of Wnt/β-catenin pathway in cooperation with PPARδ agonist (GW501516) has been demonstrated recently in the mouse model [13]. Our results affirmed the previous observations that the activation of Wnt/β-catenin signaling in early embryonic development ensures the BL competency for implantation by regulating the downstream PPARδ expression. These results also provided the evidence that Wnt dependent activation of PPARδ function is likely required for the enhancement of bovine BL hatching and implantation potential. Interestingly, our confocal imaging analysis showed a strong co-localization between β-catenin and PPARδ expression under Wnt stimulated condition (Figure 7). This observation is in agreement with the early findings [19]. It also confirmed the regulation of PPARδ expression by Wnt/β-catenin signaling.

**Figure 8:**
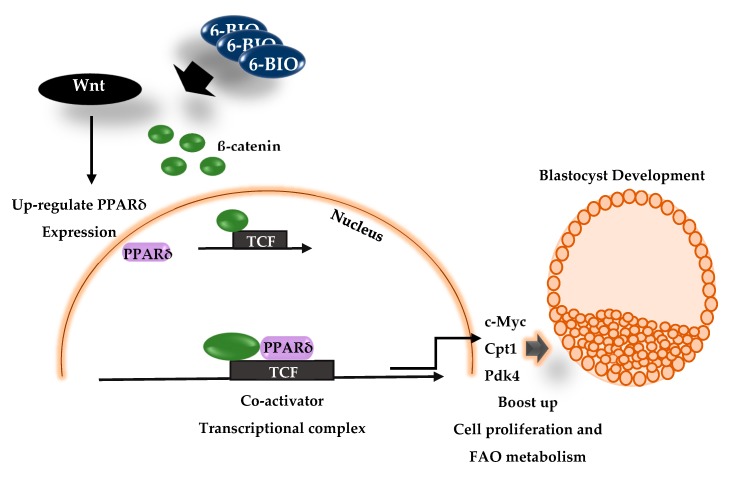
A hypothetical model for Wnt induced PPARδ expression during bovine embryonic development. Exogenous stimulation of Wnt activity by 6-Bio induced PPAR**δ** expression which co-localizes with β-catenin to make a transcriptional co-activator complex. The activator complex synergistically regulates Wnt directed genes expression and positively influence cell proliferation activity and FAO metabolism during bovine BL development and hatching.

Finally, we propose that the stimulation of canonical Wnt activity by small molecule activator 6-Bio up regulates the downstream PPARδ expression. The PPARδ in conjunction with β-catenin makes a transcriptional co-activator complex and boost the Wnt directed genes expression during the development of bovine embryos (Figure 8). In summary, exogenous activation of Wnt/β-catenin signaling by small molecule agonist 6-Bio induces the PPARδ expression and maintains the pluripotency and FAO metabolism during in-vitro bovine embryonic development. PPARδ in coordination with β-catenin transcription factor improves the hatching rate and implantation competency of BL. Our study, for the first time, unfolded the physiological significance of canonical Wnt pathway with PPARδ signaling in bovine BL development.

## Figures and Tables

**Table 1:** Effect of concentration of Wnt agonist 6-Bio on bovine blastocyst (BL) development and hatching.

Groups (nM)	Oocytes, n	Speculated Zygotes, n	Cleaved Embryos, %	Total Blastocyst, %	Hatched Blastocyst, %
Control	340	315	245 (77.8 ± 0.5) ^b^	93 (29.6 ± 0.5) ^b^	41 (44.9 ± 1.8) ^b^
6-Bio (200)	295	277	217 (78.3 ± 0.6) ^b^	94 (33.9 ± 0.9) ^c^	53 (55.9 ± 3.4) ^c^
6-Bio (300)	313	290	227 (78.3 ± 0.3) ^b^	99 (34.1 ± 1.1) ^c^	56 (55.9 ± 3.4) ^c^
6-Bio (400)	346	321	254 (78.9 ± 0.4) ^b^	121 (37.8 ± 0.5) ^d^	78 (64.3 ± 1.2) ^c^
6-Bio (500)	165	151	83 (55.0 ± 1.7) ^a^	31 (20.8 ± 1.3) ^a^	7 (22.5 ± 3.2) ^a^

^a,*b*^*p* < 0.05 with different superscript in the column indicate significant difference.

**Table 2:** Co-treatment with 6-Bio rescued the inhibitory effect of PPARδ antagonist (Gsk3787) on embryonic development to the BL stage and hatching.

Groups	Oocytes, n	Speculated Zygotes, n	Cleaved Embryos, %	Total Blastocyst, %	Hatched Blastocyst, %
Control	502	481	377 (78.5 ± 0.2) ^c^	140 (29.2 ± 0.5 ) ^c^	63 (45.0 ± 0.9) ^c^
6-Bio (400 nM)	521	496	387 (78.2 ± 0.3) ^c^	188 (37.8 ± 1.3) ^d^	115 (61.1 ± 1.6) ^d^
Gsk3787 (15 µM)	453	430	323 (75.0 ± 0.4) ^a^	69 (16.1 ± 0.6) ^a^	5 (7.2 ± 2.5) ^a^
6-Bio + Gsk3787	521	494	378 (76.7 ± 0.4) ^b^	120 (24.3 ± 0.4 ) ^b^	43 (35.5 ± 1.2) ^b^

^a,*b*^*p* < 0.05 with different superscript in the column indicate significant difference.

## Supplementary Materials

The following are available online at https://www.mdpi.com/2073-4409/9/4/923/s1, Figure S1: Mean florescence intensities of β-catenin in control and 6-Bio treated groups at different stages of embryonic development, Figure S2: Effect of dose dependent inhibition of PPARδ function on BL development and hatching rate, Figure S3: Brdu proliferation assay in Wnt agonist and PPARδ antagonist treated group, Figure S4: Quantification analysis of β-catenin and PPARδ expression, Table S1: List of qRT-PCR primers, Table S2: List of antibodies, Table S3: Effect of PPARδ inhibitor on bovine blastocyst development and hatching.
