# Supplementary material for: Role of Wnt Signaling During In-Vitro Bovine Blastocyst Development and Maturation in Synergism with PPARδ Signaling
Source: Cells. 2020 Apr 9;9(4):923. doi: 10.3390/cells9040923 (PMC7226827; doi:10.3390/cells9040923)
Supplement: Supplementary file 1 [file cells-09-00923-s001.pdf]

Supplementary Materials:

Supplementary Table S1: List of qRT-PCR primers.

Gene Name	Forward	Reverse	Accession #
GAPDH	TTCAACGGCACAGTCAAGG	ACATACTCAGCACCAGCATCAC	NM_001034034
GSK3β	AGTATACACCAACTGCCCGACT	TGAAGTTGAAGAGTGCAGGTGT	NM_001101310.1
β-catenin	AATCCGAGCTGGACCTCAATGACA	TATCATGTCCAAGCAGCCCGAGAA	NM_174637.4
Axin2	TGCTTATCTCTTCCGGACTTTC	CGCTTTGGCTACTCGTAAAGTT	XM_024979963.1
c-Myc	TTTTCTCCGTCCTCTGACTCTC	TTCCTCATCCTCTTGTTCTTCC	NM_001046074.2
CyclinD1	TGAAGGAGACCATTCCCTTG	CCACTTGAGCTTGTTCACCA	NM_007631.2
PPARα	ATATTTCCCTCTTTGTGGCTGC	ATGGTTGTTCTGTAGGTGGAGT	XM_024991367
PPARγ	GACTTCTCCAGCATTTCCACTC	ATACAGGCTCCACTTTGATTGC	NM_181024
PPARδ	AGAGCACTCACTTCCTTCCAG	GTTGCGGTTCTTCTTCTGGATT	XM_024983411
Cpt1a	GAGGGAGACTTTACACGGATGA	AGATGTATTCCTCCCACCAGTC	NM_001304989
Pdk4	GGTGATTGTTGTCTTGGGGAAA	AATTATCCATCACAGGCGTTGG	NM_001101883

Supplementary Table S2: List of antibodies used:

Target	Cat #	Manufacturer
β-catenin	ab32572	Abcam
Oct4	Sc-5279	Santa Cruz
Cdx2	AM3920119	Bio Genex
c-Myc	D84C12	Cell Signaling
Cox2	Sc-19999	Santa Cruz
PPARδ	LS-C437498	LS-BioScience
Cpt1a	Sc-393070	Santa Cruz
DAPI	62248	Thermo Fisher scientific
FITC	B2119	Santa Cruz
TRITC	A6071	Invitrogen

Supplementary Table S3: Effect of PPARδ inhibitor on bovine blastocyst development and hatching:

Groups	Oocytes, n	Speculated Zygote, n	Cleaved Embryos, n %	Total Blastocyst %	Hatched Blastocyst %
Control	391	369	288(78.0 ± 0.9)^{b}	103(28.0 ± 0.3)^{c}	45(44.4 ± 3.1)^{d}
(8μM)	340	328	253(77.1 ± 0.4)^{b}	88(27.0 ± 0.5)^{b,c}	33(37.1 ± 0.8)^{c}
(10μM)	337	331	246(74.1 ± 0.6)^{a}	82(25.0 ± 0.5)^{b}	27(32.6 ± 1.9)^{c}
(12μM)	338	317	236(74.0 ± 0.5)^{a}	57(18.3 ± 0.8)^{a}	12(20.9 ± 2.2)^{b}
(15μM)	208	199	148(74.6 ± 1.3)^{a}	32(16.2 ± 1.7)^{a}	3(9.4 ± 4.0)^{a}

^{a,b} $p<0.05$ with different superscript in the column indicate significant difference.

Supplementary Figure S1:

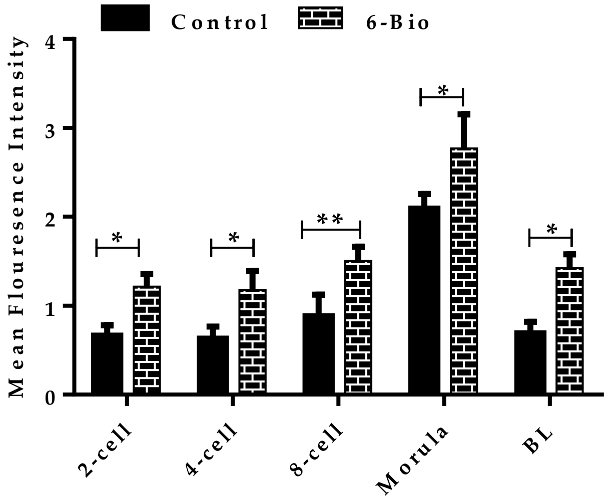

Figure S1. Mean florescence intensity of β-catenin in control and 6-Bio treated groups at different stages of embryonic development. Bar graph data of mean fluorescence intensities presented as a means ± SEM from three individual sets of experiment, including n = 20 BLs per group in each replicate. * $p < 0.05$; ** $p < 0.01$; indicates significant difference.

Supplementary Figure S2:

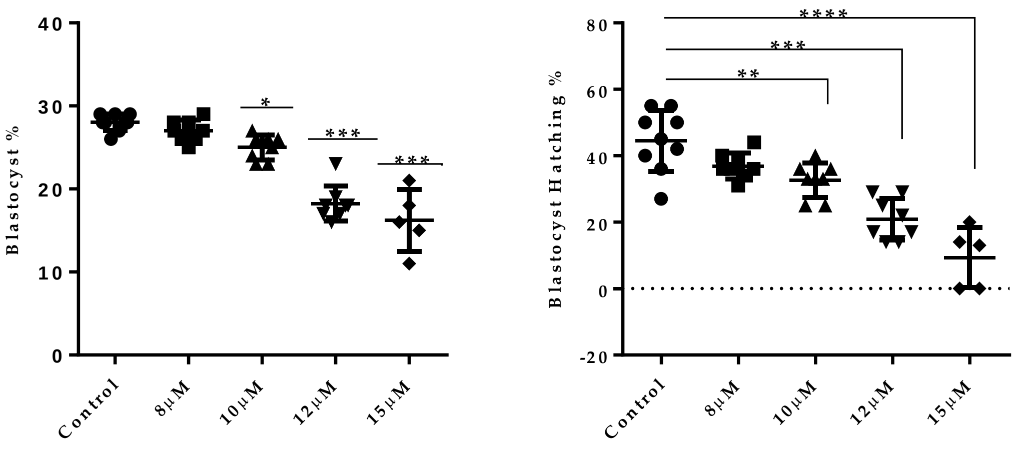

(a) (b)

Figure S2: Effect of dose dependent inhibition of PPAR function on BL development and hatching rate. (a-b) Graphical data shows the % of bovine blastocyst development and hatching during different concentration of Gsk3787. Data presented as a means ± SEM from three individual sets of experiment, including n = 20 BLs per group in each replicate. * $p < 0.05$; ** $p < 0.01$; *** $p < 0.001$; ; **** $p < 0.000$; indicates significant difference.

Supplementary Figure S3:

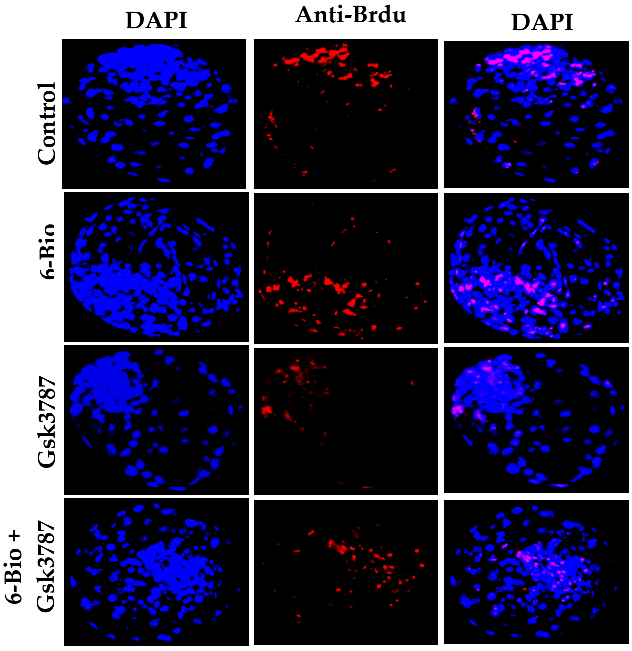

(a

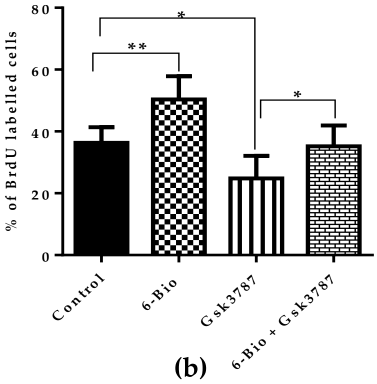

(b)

Figure S3: Brdu proliferation assay in Wnt agonist and PPARδ antagonist treated group. **(a)** Immunofloresence staining showing the anti-Brdu labelled cells in different treatment groups. **(b)** Graphical data shows the % of Brdu labelled cells in different treatment groups. Data presented as means ± SEM from three individual sets of experiment, including n = 20 BLs per group in each replicate. * $p < 0.05$; ** $p < 0.01$; indicates significant difference.

Supplementary Figure S4:

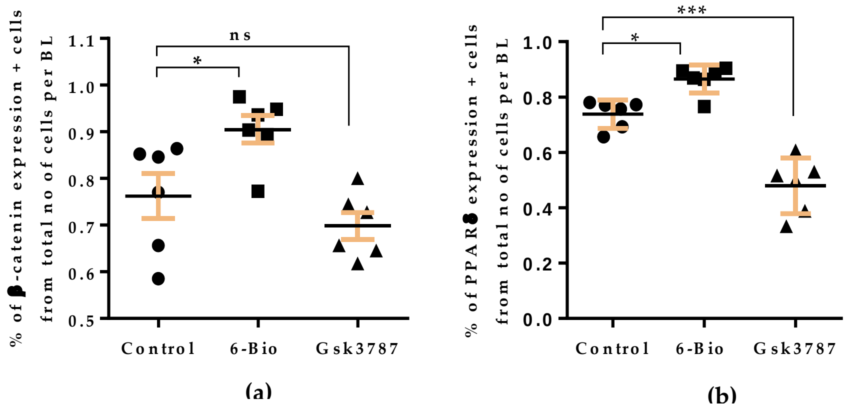

Figure S4: Quantitative analysis of β-catenin and PPARδ expression. **(a-b)** graphical data represents the percentage of β-catenin and PPARδ expression positive cells from total number of cells per BL in each group. ± SEM from three individual sets of experiments (n = 20 BLs). ns,non-significant, * $p < 0.05$; *** $p < 0.001$ indicates significant difference.
